# Addressing privacy concerns for mobile and wearable devices sensors: Small-group interviews with healthy adults and cancer survivors

**DOI:** 10.1016/j.pecinn.2022.100022

**Published:** 2022-02-06

**Authors:** Grace Ellen Brannon, Sophia Mitchell, Yue Liao

**Affiliations:** a Tenure-Track, Department of Communication, College of Liberal Arts, University of Texas at Arlington, 700 West Nedderman Drive, FAB 118, Arlington, TX 76019, United States of America; b Department of Communication, College of Liberal Arts, University of Texas at Arlington, United States of America; c Tenure-Track, Department of Kinesiology, College of Nursing and Health Innovation, University of Texas at Arlington, 500 West Nedderman Drive, MAC 147, Arlington, TX 76019, United States of America

**Keywords:** Privacy, Wearable sensors, Technology, Interviews

## Abstract

**Objective:**

Mobile and wearable sensor technology is increasingly common and accessible. The aim of this study was to explore individuals' perceptions and acceptability of mobile and wearable sensors, as well as concerns.

**Methods:**

Purposive sampling was used to recruit non-patient adults (*n* = 22) and cancer survivors (*n* = 17) for face-to-face and virtual small-group interviews. Reflexive thematic analysis of the data focused on privacy concerns.

**Results:**

Participants reported that privacy was generally not a concern for sensor adoptions for physical activity health interventions except for health insurer access.

**Conclusion:**

The patient perspectives as reported in the findings illustrate the need for transparency between potential adopters and users of mobile and wearable devices and health care practitioners, as well as secure privacy policies for health insurers.

**Innovation:**

Older adults often are perceived as unwilling to adopt mHealth technologies for many reasons, including privacy concerns. This study examined an important patient population, cancer survivors, who are often overlooked yet may benefit from targeted health interventions using mHealth technologies, and compared their responses with a non-patient population for prevention purposes. Our findings suggest that one's lived health experiences (cancer survivorship) are more influential than one's age in adopting mHealth technologies.

## Highlights

• Privacy concerns for mobile and wearable device technology interventions are minimal
• Both healthy adults and cancer survivors reported similar (lack of) concerns
• Hesitations included health insurers access and employers

## 1 Introduction

**Fig. 1:**
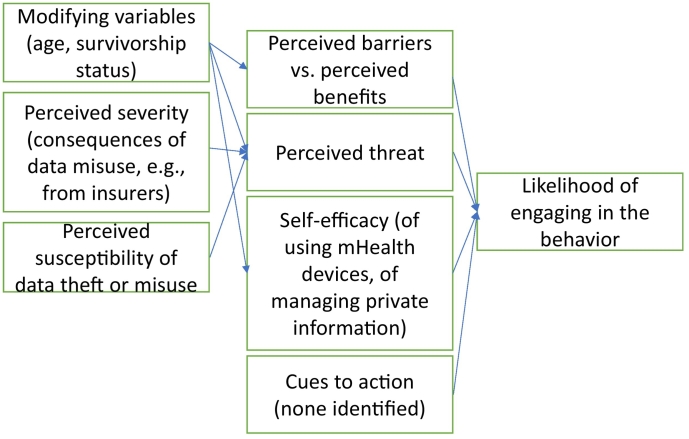
Mapping main findings onto the Health Belief Model.

Mobile and wearable devices provide great insight for individual health and are increasingly common given the prevalence of smartphones and other portable devices [1]. Mobile and wearable devices include all sensors, smartphones, smartwatches, as well as smartphone applications, that communicate a user's health information to them. Early generations of mobile and wearable devices focused on tracking physical activity levels and heart rate [2]. More recently, major public health challenges like diabetes management are a primary focus [3]. In tackling these challenges, research reveals several important trends for optimal performance and acceptability of wearable devices: flexibility, transparency, adhesion property, water repellency, and biocompatibility [4,5] – with the commonality of each of these being the physical features of the technology. Less is known, however, regarding potential users' perceptions of acceptability for the non-physiological aspects of mobile and wearable devices sensors specifically related to privacy concerns. (See Fig. 1.)

Most recent research contrasts digital health technology perceptions as they change over time for one specific group (i.e., cancer survivors), rather than contrasting between patient and non-patient groups [6]. For at-risk patient populations (e.g., older adults with chronic diseases, such as cancer survivors) who likely have greater mHealth technology hesitations, this is a particularly important area of research especially within the larger context of an increasingly aging population [7]. Research on these older adults' perceptions of mHealth focuses on how physical activity energy expenditure and all-cause mortality are associated [8] and general perceptions of wearable activity trackers [9]. Therefore, this study aims to bridge this gap in the literature by examining perceptions and concerns of privacy related to wearable sensors using Health Belief Model (HBM) as the theoretical lens [10].

### 1.1 Mobile health technology

Mobile and wearable sensors can be used to complement in-person visits to health care practitioners, and are expected to reduce the burden on health care personnel [11]. Recent qualitative research shows that activity trackers such as Fitbits, are perceived positively related to four factors: sleep hygiene, motivation, discretion, and accountability [12]. Other wearable technologies, like continuous glucose monitors, show great promise for providing users with real-time data, and with proper training, provide important data for users to see the links between specific behaviors (e.g., physical activity or food consumption) and short-term physiological health outcomes (e.g., in-range or low or elevated glucose levels) [13]. One author claimed that the knowledge obtained from the data allows individuals more control in progressing towards their health goals [14]. They also are expected to improve productivity and efficiency in health care encounters [13].

Mobile and wearable devices are not currently used to their full potential, however. One oft-cited concern is that of user privacy. Privacy concerns encompass information privacy, data sharing, autonomy, consent, ownership, data access, and data valuation [15]. For example, an individual's health information gathered or retained or shared via a mobile application may be at risk if the product is not properly digitally secured, or if the company falls victim to a hack [16]. Some organizations have moved towards an “opt in” approach with their data, allowing the user greater opportunity to determine who and how their data is accessed and used [17]. Ambivalent users tend to have high levels of both perceived benefits and privacy concerns simultaneously, yet these same individuals also tend to have lower continued intention of use [18]. One recent study used a one-item question asking about privacy concerns, with participants providing ratings for an activity tracker, a CGM, and a smartphone app, and reported that privacy concerns were low [53]. Further, another burgeoning area of related research examines personalized messaging and feedback [19]. Identifying population-specific hesitations or concerns provides value for healthcare practitioners and researchers for designing health interventions. Disease prevention and health promotion efforts for non-patient populations are likely different than disease management messaging for patient populations. Specifically, different population groups (e.g., a cancer survivor compared with a person without a cancer diagnosis) might have different needs and face different health challenges, and therefore, their attitude and usage of mobile and wearable devices sensors could vary. For example, cancer survivors face both short-term and long-term effects of chemotherapy regimens that others are not at risk for, along with increased risks of cardiac disease due to radiation treatments [20]. These specific health challenges may increase a cancer survivor's need over the general population's for supportive care, including emotional and psychological support. Mobile and wearable devices may be one method of providing supportive care to this population.

### 1.2 Health belief model

HBM is one of the most widely applied theories of health behavior, having been adapted for many culturally and topically diverse contexts [21,22]. HBM posits six specific constructs that predict health behaviors: risk susceptibility (if the individual feels at-risk), risk severity (consequences), benefits to action (taking action would reduce either or both severity and susceptibility or lead to other positive outcomes), barriers to action (potential negative attributes related to the health action), self-efficacy (ability to complete the behavior of interest despite the barriers), and cues to action (internal or external factors in one's environment) [10,23]. The HBM model has been used to recommend best practices for older adults regarding improving their physical activity levels [24]. Patient education delivered through mHealth methods using HBM-influenced materials can improve physical activity levels among pregnant women [25], yet these same strategies have yet to be applied in the cancer survivor context.

HBM constructs are associated with respondent intentions to adopt mobile health applications [26,27]. In the health information/privacy context, perceived susceptibility is where one feels that their risk of having their health data stolen may be high. Perceived severity is how serious the consequences of data theft may be. Previous research has examined children's virtual privacy using HBM, finding that increased privacy concerns were associated with decreased exposure to online risks [28]. Perceived benefits of sharing data with health care providers via health technology may include having the data to make more informed health decisions, leading to a specific health outcome [27]. Perceived barriers may include time or money or limited understanding of how one's private health information may be used. Self-efficacy refers to how capable one feels that they can perform a specific behavior, such as using the wearable sensors and managing one's own privacy on apps. Finally, cues to action could include seeing a social media advertisement of a specific health app that might cue the user to performing a specific action such as providing a health care provider access to one's data. One critique of HBM is that the model fails to specify the order of variables [23]. Recent research investigated this limitation, finding an underlying hierarchy with self-efficacy as a moderator, barriers as a mediator, and barriers to benefits as a causal chain [29].

Recently, calls for more research center on the reoccurring analysis and criticism surrounding personal information and data protection given the uptick in wearable technologies [17]. Further, calls exist specifically for empirical research examining the inconsistencies, contradictions, negotiations, and/or rejections of mobile and wearable sensors [30]. Increasing understanding for health promotion purposes in different populations is important for designing health interventions. Since elements of health behaviors are partially responsible for an individual's motivation to engage in health-promoting behaviors like physical activity, and these may be complicated by the privacy concerns related to wearable devices, and considering these recent calls for research, the following research questions are posed.

RQ1 What privacy concerns do individuals have regarding mobile and wearable device adoption?

Previous research leads us to believe that privacy concerns are important, particularly for health promotion research. A recent study examined perceptions of COVID-19 contact tracing apps using HBM measures, finding that perceived barriers, particularly privacy concerns, negatively predict app uptake intention [26]. Other than this study, however, little is known about how privacy elements may be driven by HBM constructs [31], which is often the theoretical basis for larger scale empirical research [32]. Privacy is also more complex than simply functioning as a barrier for health behaviors. Research on the privacy paradox (balancing the need to disclose and the need to conceal) demonstrates the complexity of privacy [33]. Therefore, we propose the following RQ as a foundational attempt to move towards improved health intervention designs:

RQ1b How might these privacy concerns be mapped onto the Health Belief Model?

## 2 Methods

### 2.1 Study design

To examine people's perceptions and concerns of privacy related to wearable sensors, we used an exploratory, cross-sectional qualitative small group interview approach. The Institutional Review Board Name (at MD Anderson) approved the study protocol (MDA 2018-0299). Signed informed consent was obtained from all participants.

### 2.2 Recruitment and eligibility

Insufficiently active, overweight/obese adults (22 non-patients [aged 25–52] and 17 cancer survivors [aged 50–74]) were recruited to participate in the study. Of the participants, the majority (*n* = 36) were females and three were males. The former group were recruited from participants in previous research studies and programs through which they were exposed to wearable sensors. The latter group included stage 0-III breast or colorectal cancer survivors over the age of 18 were identified from MD Anderson's tumor registry. Participants were contacted by email with a brief study description. Those who expressed an interest were invited to fill out an eligibility screener either through an online survey or via phone.

To be eligible for the study, all participants were required to be (1) adults over 18 years of age, (2) overweight or obese (i.e., body mass index ≥25 kg/m^2^ based on self-reported height and weight), (3) insufficiently active (i.e., engage in less than 150 min of moderate-intensity physical activity per week in the past month based on self-report), (4) able to walk one block without pain or discomfort, and (5) able to speak, read, and write in English and the individuals reporting that they were cancer survivors additionally were required to have completed adjuvant therapy (i.e., chemo and/or radiation therapy). Participants were excluded if they reported health issues limiting their physical activity levels or were on dialysis.

### 2.3 Procedure

Eligible participants were scheduled for in-person group meeting based on survivorship status or non-patient adult status at MD Anderson. All participants signed an informed consent prior to the start of the group discussion. A moderator led the small group interviews using a theory-influenced interview guide [34,35]. The interviews were based on a semi-structured interview form, and topics encompassed the participants' current physical activity levels, their perceptions of two types of wearable sensors (i.e., an activity tracker like Fitbit and a continuous glucose monitor like Dexcom and Freestyle Libre), related sensor features and apps, and their general experiences, perceptions, preferences, and opinions on using the sensors. Each of the five sessions lasted 60–90 min and were audio-recorded. Sessions were transcribed verbatim by a professional transcription company.

Beginning in March 2020, all in-person groups ceased due to the COVID-19 pandemic. After amending the study protocols, group meetings resumed via an institutional Zoom account. These practices align with best practice recommendations regarding COVID-19 and qualitative research [36]. Excepting the group communication medium, study procedures remained the same. Participants received compensation in the form of a $15 gift card.

### 2.4 Analysis

Reflexive thematic analysis using the process of data collection, processing, and analysis was encompassed by six steps [37,38]. Participant responses were the unit of analysis. The first step involved data familiarization, in which the small group interview transcripts were read in their entirety on Dedoose by the principal investigator, a co-investigator, and a graduate research assistant. During this step, the review of transcripts provided understanding of the breadth of the data, and the research team referred to the initial interview guides as needed to provide context. Next, major content areas were categorized in open coding, followed by axial coding in which initial coding categories and subcategories were identified [39]. Transcripts were then reviewed again to identify specific examples illustrating the research themes during the selective coding process. Analyzing these codes preceded the creation of themes. The transcripts were reread yet again after the codebook was created to ensure that all relevant information was gathered, and the data fully analyzed. Two of the three researchers were involved at all steps of analysis, with the third involved for discussions if needed. During the interviews, the interviewer checked for participant understanding and asked clarifying questions, which allowed the participants chances to challenge potential misunderstandings of the researchers. This was done throughout as a method of validity and as a data trustworthiness check [39,40]. The codebook provided an audit trail [41]. Validity was assessed using the four primary criteria of credibility (accurately interpreting participant meaning, which was achieved through participant checks), authenticity (hearing from different voices from the different participant groups), criticality (appraising all aspects of research from design through analysis), and integrity (self-reflexivity) [40]. Reflexively speaking, none of the research team has had cancer, and one (who did not interact with participants) wears a continuous glucose monitor – these lived experiences affect researcher interactions with the data. As qualitative research is subjective, the analysis is situated from the interaction of the data, research positionality, and the context of the research itself [38]. The subsequent section provides exemplars from the participants, obtained from the small group interviews, to illustrate the identified themes. Assigned pseudonyms are used to quote participants in this manuscript.

## 3 Results

### 3.1 General privacy concerns

Participants were asked to describe their general privacy concerns about using wearable sensors like Fitbit or CGMs. Themes regarding the key concerns encompassed information privacy, data sharing, data access, and ownership, with clear links to HBM constructs, including severity, susceptibility, barriers, benefits, and self-efficacy. The primary similarity between the cancer survivor and non-patient groups was that both groups indicating fear of their health data being shared with their health insurance companies. Differences between the groups included 1) cancer survivors being freer with their health information, with looser metaphorical boundaries around their information, and 2) cancer survivors engaging in greater levels of data access (interacting with their own data virtually and allowing their health care providers virtual access as well) compared with non-patients.

### 3.2 Perceived benefits

Most of the participants indicated they were unconcerned about their information privacy, with the perceived benefits of having their information shared with others outweighing the perceived barriers. For example, Lindy (non-patient) said, “[I have] no real privacy concerns. Everything else is using our data, so what's one more thing?” Two other participants in Lindy's interview group agreed without hesitation. Given that so many people share a breadth and depth of health information online already, the responses to this question were generally unsurprising. Participants therefore did not feel their health information was at risk of theft, thereby indicating low levels of perceived susceptibility. Jackie, non-patient, stated that, “I don't necessarily have any concerns with privacy,” and instead focused on how interesting it might be to have biological data like glucose, or even blood pressure and heart rate, available and easily trackable. When probed to discuss that comment further, Jackie said that she'd like to know more about her own biological data for educational purposes “just so that I can learn”, especially given her family's history of hypoglycemia. Knowing that a third party had access to this same data did not deter her from believing the perceived benefits of having the data herself were likely. Other participants had similar stories – the potential biological data provided possibilities for increasing knowledge or connection with family members.

### 3.3 Perceived barriers

Ownership related to data sharing was another story entirely, however, and was considered a barrier for some participants. Participants felt that their information being out (perceived severity) beyond their control could be problematic. Knowing who has access to one's health information, and the implications for how they might share or use their health information, was of great importance for several participants, but the topic was more common for non-patients than cancer survivors. Of note for both groups was that participants indicated that they were generally unconcerned about privacy, except for third parties like insurance companies having their information, as they could “affect our lives” (Laura, non-patient) “by raising everything sky high” (Lindy). Bailey (cancer survivor) mentioned that she was particularly concerned that the data from a wearable monitor could be used to deny insurance coverage. This concern was echoed by multiple participants across groups. Lucille (non-patient) stated, “Insurance companies are going to raise your rates because you're not exercise[ing], or your glucose is too high, or that kind of thing. That would be a concern.” Anne (cancer survivor) mentioned that she didn't care if someone saw “how bad I am” but wanted to know what might happen for an action plan, afterwards. Bailey said, “I feel like it doesn't bother me to have people know or to have professionals that are going over the data. That doesn't bother me.” Each of these participants, as well as their fellow interview group members, did not feel that their health information, if considered negative, would be managed well. Further, these statements show the need for transparency in privacy practices. Knowing who has access wasn't enough for the participants, but also knowing what the receiver might do with the information, and how that might directly affect the participant (i.e., by access to health care coverage, cost of coverage, or coverage of service status). Perceiving both unwanted access to one's private health information, as well as potential dangers associated with that (i.e., the expenses from potential increases to cost of health insurance) were the few barriers participants discussed.

### 3.4 Self-efficacy

Data sharing was another primary theme and was characterized by a tension between perceived benefits and perceived barriers, with the underlying cause likely one's self-efficacy. Other participants used their individual health experiences to provide context around their privacy conceptualizations (describing higher levels of self-efficacy regarding using their health data appropriately). For example, Addison (cancer survivor) described her thoughts in the context of her previous cancer treatment,

I think before the cancer me, I would have said yeah. Because now you get phone calls and they want to–people call you on the phone and I'm like, “How do you know all this stuff? How do you know?” I hang up, you know. But the new me, because now it's a new me, I'm a new person now, right, I don't find that I need to hide anything. I feel that it's okay for it to be open and out because it's going to help somebody else, I think, too. And I don't feel I have anything to hide because the more I speak, the more I learn, because somebody is doing something that I need to do, that if I don't speak and if they don't know, they can't help me, you see?

Bailey felt similarly, saying that she only cared about protecting their “banking account and my social security number” but that any health information, particularly because their cancer treatment was at a teaching hospital, was more helpful for others to have as it can be learned from, “And I feel like that I'm a teacher, so if there's something that somebody can learn from what we do or we can be a part of, then I'm happy to be a part of that. I'm not too worried about whether or not somebody finds out I have cancer.” For these participants, it was again important that the intent behind obtaining one's private health information, particularly if communicated clearly, was vital. Being more open was almost easier, or at least, more understandable, if the receiver had a reasonable rationale for knowing the information. Based on the differences between the non-patient and the cancer survivor groups, therefore, this study presented us with the unique opportunity to examine privacy at both the disease prevention and the disease management/control perspectives.

In general, most of the participants felt that privacy concerns, as minimal as they were, did not decrease their perceptions of acceptability regarding the mobile and wearable devices. In fact, the survivors perceived the sensors positively despite the identification of other potential barriers to using the mobile and wearable devices (i.e., the subcutaneous device insertions) and potential privacy issues identified within the groups and by the moderator. Participants did identify seeing news stories about identity or personal information being leaked from a big consumer product company, making them more worried about privacy and thus not willing to share (thereby using avoidance as their action).

## 4 Discussion and conclusion

### 4.1 Discussion

Using Health Belief Model as the theoretical framework, participant responses illuminated several important issues related to information privacy, data sharing, data access, and ownership. The first research question focused on privacy concerns individuals have regarding mobile and wearable device adoption. RQ1b examined how these specific privacy concerns, as situated within the themes of information privacy, data sharing, data access, and ownership, might be mapped onto HBM constructs. The following section details the privacy concerns, while tying in the key themes of the study's findings with HBM constructs to answer both RQs.

First, participants of both groups (all of whom were overweight or obese) perceived that they needed to engage in PA more, and that remotely delivered interventions are promising methods for them to do so with benefits of the intervention outweighing the risks of privacy concerns. In fact, participants seemed to encourage the use of mHealth technologies for this purpose, which contradicts previous research showing that mHealth-delivered interventions may be effective in increasing physical activity in older adults in the short term [42]. However, research shows that discontinuance may occur for many reasons, over multiple periods of time (i.e., initially, over weeks, or even months) [30], and therefore future research should investigate the long-term effects of using CGMs in particular in this manner. Participants, regardless of patient status, were open to mHealth. Our results showed that mHealth tools can be a viable strategy for health promotion as the non-patients indicated their willingness to share their personal data with intervention programs that aims to promote healthy lifestyle. Therefore, our findings show that people are actually very open about prevention efforts, which is excellent news for health promotion intervention programs. And, while all participants were overweight or obese, it could be that they did not feel high levels of perceived severity or susceptibility related to their health status. There further seemed to be a disparity between non-patients and cancer survivors, as cancer survivors seemed to perceive the mHealth technologies more positively based on their cancer experiences. It may be that their healthcare experiences as cancer survivors increased their self-efficacy, a vital component of the HBM. As recent researchers developed a HBM-influenced scale, future research should quantitatively examine these processes [43].

Second, participants in the present study indicated that data usage transparency was of particular importance. With machine intelligence being deployed more frequently in health care settings, this is a particularly timely finding [44]. Yet, a recent empirical study examining mobile health application privacy policies and practices found that 41% of the apps did not have a privacy policy in place that would inform users about how and when the information shared over the app would be collected, retained, or shared with third parties [45]. These practices could potentially limit trust in organizations, particularly with the recent prolific hacks, which in turn may lead to decreased use of specific apps or technologies. Recent research demonstrates people perceive a need for privacy-preserving aspects in digital solutions [26,46]. Health interventions in the future should take care to provide detailed privacy policy practices for potential participants to alleviate the participants' valid concerns. However, these same privacy policy practices should be communicated as clearly and succinctly as possible, particularly as low levels of health literacy are an issue in the United States and increased levels of health literacy were linked in one systematic review with deliberate choices about physical activity [47].

Third, and to the point of this study, participants in both groups perceived both benefits and barriers related to privacy. Contrary to past research, autonomy, consent, ownership and data valuation were not prevalent in this research [15]. This may be because, for at least the cancer survivors, their lived experiences skewed their perceptions of having consent or data valuation. Other past research shows mixed findings regarding privacy concerns. First, some research shows that many individuals feel concerns about privacy, with negative influences from coping appraisals (i.e., response efficacy and self-efficacy) and positive influences from perceived vulnerability and perceived severity [48]. The present research qualitatively provides further evidence for this growing body of evidence. Specifically, participants were concerned about being discriminated against by employers or insurance companies if their health information is accessible by these constituents. As HBM research on mHealth adoption shows that barriers are strongest if they are expensive, dangerous, unpleasant, time-consuming, or inconvenient, and dealing with health insurance companies can be a combination of several of those, our finding aligns with previous HBM research [49,50]. Yet, with that exception, many participants in the present study did not have hesitations about sharing their private health information, which contradicts recent research [26]. This study therefore aligns with burgeoning research areas and extends other research by finding that privacy concerns towards mobile and wearable devices are minimal (AUTHOR, year). Of particular interest was one participant's recognition that individual health circumstances may affect those beliefs. Having specific health experiences (i.e., a diagnosis of cancer) clearly influenced the participant's perceptions related to privacy concerns. Future research should quantitatively examine the extent to which individual characteristics (e.g., demographic variables or psychological characteristics) may predict how perceived barriers and benefits of mobile and wearable sensors may influence sensor adoption behaviors.

Two specific implications can be understood because of this study's findings. First, participants were generally unconcerned about their private health information being shared, except for the information being shared with their insurance companies. This hesitation is likely at odds with health insurers' recent pushes to enroll and involve employees on specific insurance plans in health management courses (i.e., HingeHealth or Livongo). Insurance companies should clarify how certain health information obtained from these types of interventions may be used, and further, how the information obtained from participant involvement in these interventions may affect insurance premiums. Second, health intervention researchers need to be cognizant of these hesitations, as they may preclude many individuals from participating in research studies. For marginalized groups who already have a pre-existing mistrust of the healthcare system (i.e., people of color or of low socioeconomic status), the addition of perceived insurance company misconduct could potentially keep many from participating, both hurting their health at the individual level plus at the societal level.

While strengths of the present study included perspectives from both cancer survivors and non-patient adults, there were some limitations. First, the small groups interviews were cross-sectional, which limits potential causality claims. Future research should continue to examine the differences and similarities between groups so if need be, messages surrounding privacy concerns may be tailored to the specific audience. Second, all participants were recruited from a large metropolitan area in South Texas, limiting potential participant variances based on geographical location. Third, health insurance status (private, public, not insured) was not collected, so claims cannot be made to differentiate these groups. Yet, as insurance status can affect the types of health technologies one has access to, this limitation should be addressed for future studies. Fourth, this study only examines a small portion of the Health Belief Model. Future research should examine other components; for example, investigating how text messages or notifications related to the mobile and wearable devices may function as cues to action. Fifth, the two participant groups varied greatly in age ranges, and we did not collect education level from participants. This may lead to biased results and interpretations. Future research should examine more homogenous groups for comparisons, particularly as people from historically marginalized racial groups face health disparities. Digital health disparities is an emerging topic that merits further investigation; examining the privacy concerns by racial group may provide insights to communities who historically have been excluded from health research [51]. Finally, while the research team strove to consider our own bias within the reflexive thematic analysis process, we must acknowledge that our own subjectivity can affect the data interpretation, and subsequently, the generalizability of the findings. Future research could extend our study by using quantitative measures to examine privacy beliefs in a larger participant population.

### 4.2 Innovation

We specifically focused our research on the experiences of an important and increasingly growing demographic, that of older cancer survivors, while using middle-aged non-patient adults with similar activity and BMI status as a comparison group. While privacy concerns related to mHealth technologies are generally established, particularly for older adults, our findings demonstrate how one's health condition or status as a cancer survivor can dramatically change one's perspectives on privacy and information sharing. For the present study's non-patient population, there was greater hesitation towards mHealth technology. Therefore, for preventive efforts utilizing digital health tools, researchers might need to first assess and address these individuals' potential privacy concerns. Subsequently, it is important for health care providers to fully understand their patients' medical history, particularly when attempting to motivate the patient towards specific health behaviors (i.e., physical activity). Future research should also examine older adults with other types of conditions (e.g., chronic diseases like diabetes) to investigate if other lived health experiences dramatically shift one's perspectives towards mHealth technologies specific to privacy concerns.

Further, our study shows how our cancer survivor/patient participants, who were older adults aged 50–74 years, perceive digital health as useful and potentially helpful. This is in contrast with a common assumption that older adults perceive mHealth technology as too difficult to use (specifically by having low self-efficacy) while also perceiving many barriers for usage and few perceived benefits [52]. Health care providers should therefore take care to acknowledge that age alone is not indicative of one's willingness to adopt digital health technologies, even among older generations who tend to be more private. Health care providers should continue to ascertain each patient's willingness to adopt mHealth as a complementary part of their treatment at an individual level. Providing personalization options through different types of apps (perhaps rated by usage of daily involvement versus moderate/weekly involvement) is one potential pathway through which this can be accomplished.

### 4.3 Conclusion

From the patient perspectives provided, we identified that privacy concerns surrounding mobile and wearable device technology interventions for improving physical activity health outcomes are minimal, and for some, nonexistent. Perceived susceptibility and severity to data issues were thereby low. For both the cancer survivors and the non-patient groups, the exception to the lack of privacy concerns focused solely on insurance companies, particularly related to insurance premiums. Therefore, while barriers were perceived related to data sharing, the benefits tended to outweigh the potential costs. Data sharing was another primary theme and was characterized by a tension between perceived benefits and perceived barriers. Further, both groups reported similar (lack of) concerns regarding mobile and wearable sensor technology. However, cancer survivors indicated they were less cautious than the non-patients indicated in providing their health information given their previous health care experiences; these experiences provided the cancer survivors with higher self-efficacy. The survivors also described engaging in accessing health data more frequently than the non-patient groups. Knowing the differences between cancer survivors and non-patient adults may help health care researchers and providers in tailoring interventions with personalized messaging for each group to encourage physical activity. Future research should continue to examine perceptions of those from marginalized groups (i.e., people of color or low socioeconomic status), as well as those from a variety of health insurance statuses.

## Declaration of Competing Interest

The authors declare that they have no conflicting or competing interests to report.
